# Sulforaphane Inhibits HIV Infection of Macrophages through Nrf2

**DOI:** 10.1371/journal.ppat.1005581

**Published:** 2016-04-19

**Authors:** Andrea Kinga Marias Furuya, Hamayun J. Sharifi, Robert M. Jellinger, Paul Cristofano, Binshan Shi, Carlos M. C. de Noronha

**Affiliations:** 1 Center for Immunology and Microbial Disease, Albany Medical College, Albany, New York, United States of America; 2 Division of HIV Medicine, Albany Medical Center, Albany, New York, United States of America; 3 Albany Medical College, Albany Medical Center, Albany, New York, United States of America; 4 Albany College of Pharmacy and Health Sciences, Albany, New York, United States of America; University of North Carolina at Chapel Hill, UNITED STATES

## Abstract

Marburg virus, the Kaposi's sarcoma-associated herpesvirus (KSHV) and Dengue virus all activate, and benefit from, expression of the transcription regulator nuclear erythroid 2-related factor 2 (Nrf2). The impact of Nrf2 activation on human immunodeficiency virus (HIV) infection has not been tested. Sulforaphane (SFN), produced in cruciferous vegetables after mechanical damage, mobilizes Nrf2 to potently reprogram cellular gene expression. Here we show for the first time that SFN blocks HIV infection in primary macrophages but not in primary T cells. Similarly SFN blocks infection in PMA-differentiated promonocytic cell lines, but not in other cell lines tested. siRNA-mediated depletion of Nrf2 boosted HIV infectivity in primary macrophages and reduced the anti-viral effects of SFN treatment. This supports a model in which anti-viral activity is mediated through Nrf2 after it is mobilized by SFN. We further found that, like the type I interferon-induced cellular anti-viral proteins SAMHD1 and MX2, SFN treatment blocks infection after entry, but before formation of 2-LTR circles. Interestingly however, neither SAMHD1 nor MX2 were upregulated. This shows for the first time that Nrf2 action can potently block HIV infection and highlights a novel way to trigger this inhibition.

## Author Summary

Nrf2 turns on anti-oxidant genes in response to pharmaceuticals like oltipratz, environmental agents like heavy metals and cigarette smoke, endogenous agents like nitrous oxide and nitro-fatty acids and even plant products like sulforaphane (SFN) and epigallocatechin gallate (EGCG). An increasing body of work is showing that some viruses activate and benefit from Nrf2. In this work we tested the impact of Nrf2 on HIV. We used SFN, abundant in cruciferous vegetables and often used as a dietary supplement, to activate Nrf2. Here we show, for the first time, that in immune cells isolated from donor blood, SFN halts HIV infection in macrophages, but not in T cells. We further show that upon SFN treatment the virus is blocked after it has transcribed its RNA-encoded genome into DNA, but before this genetic material is inserted into host chromosomes. Importantly this block is indeed dependent on Nrf2. Interestingly, Nrf2 does not activate recognized anti-viral genes. Thus, unlike viruses recently found to benefit from Nrf2 activation, HIV can be blocked by its activation. This highlights the opportunity to activate a heretofore unrecognized anti-viral function by triggering an antioxidant response with a common dietary component.

## Introduction

Highly active anti-retroviral treatment (HAART) is saving countless lives, however its application is accompanied by high financial costs, the emergence of resistant viruses and short- and long-term side-effects. A better understanding of how to activate cellular anti-viral defenses promises new therapeutic alternatives to overcome these limitations and to support prevention and cure strategies.

Here we show for the first time that sulforaphane (SFN), a natural product recognized for its health benefits, blocks HIV infection in macrophages. These cells play a critical role in HIV infection and pathogenesis, forming long-lived viral reservoirs [1, 2] and carrying virus into restricted compartments like the brain [3]. Most monocytes, precursors of macrophages, are largely refractory to HIV infection until they differentiate to replenish the macrophage pool [4, 5]. A small but specific subset of these cells however may be readily infectable, even in HAART-treated patients [6–9]. Infected macrophages have been observed in asymptomatic, untreated patients [10] and in HAART-treated individuals [11]. Other work, while not directly probing infection *in situ*, shows that rectal and vaginal macrophages share markers with HIV-susceptible macrophages [12, 13]. Importantly, macrophages can powerfully increase T cell infection [14] and may thereby exacerbate T cell depletion in infected individuals.

SFN is generated in cruciferous vegetables after mechanical damage, like chewing, causes admixing of glucoraphanin with the enzyme myrosinase. It reaches various tissues after consumption where it maintains biological function [15]. In humans SFN is absorbed in the gut and peak plasma levels are achieved within 90 minutes [16]. In mice SFN can reach the prostate after ingestion [17, 18] where biological activity is maintained [19–21].

SFN is a powerful mobilizer of the transcription factor nuclear factor erythroid 2-related factor 2 (Nrf2) [22–24]. Nrf2, a master regulator of the antioxidant response [25, 26], is held in check by its constitutive degradation through CUL3 ubiquitin ligase complexes using the protein KEAP1 as a substrate adaptor. SFN and other Nrf2-activators modify KEAP1 to prevent engagement of Nrf2 [27]. As Nrf2 accumulates, it translocates into the nucleus, partners with musculoaponeurotic fibrosarcoma protein (Maf) and initiates transcription from antioxidant response elements (ARE). The consensus Nrf2 binding motif, TGACTCAGCA, was derived from known anti-oxidant response genes and Nrf2 binding was reconfirmed in chromatin immunoprecipitation and transcription analyses [28]. Nrf2 activates several types of genes [29] which predominantly encode proteins that counter oxidizing conditions [30].

Three viruses, Marburg virus [31, 32] Kaposi's sarcoma-associated herpesvirus (KSHV) [33] and Dengue virus [34] trigger Nrf2 activation. KSHV and Dengue virus appear to cause Nrf2 mobilization indirectly. Marburg virus however boosts Nrf2 levels directly with a protein designated VP24. This protein assembles with Keap1 to, like SFN, hamper engagement of Nrf2 and thus Nrf2 turnover [31, 32]. All three viruses benefit from increased Nrf2 expression [31–34].

The impact of SFN and Nrf2 on HIV-1 and HIV-2 infection, in primary cells, has not been determined. Here we show that SFN, acting through Nrf2, triggers a strong block against HIV infection in myeloid-derived cells including primary macrophages, but not in primary T cells. SFN blocks infection after reverse transcription but before the formation of 2-LTR circles. Together this work shows that, in contrast to Marburg, Dengue and KSHV, HIV is blocked through Nrf2 and sets the stage for discovering the mechanism underlying this restriction.

## Results

### SFN blocks HIV in primary macrophages but not in primary T cells

**Fig 1:**
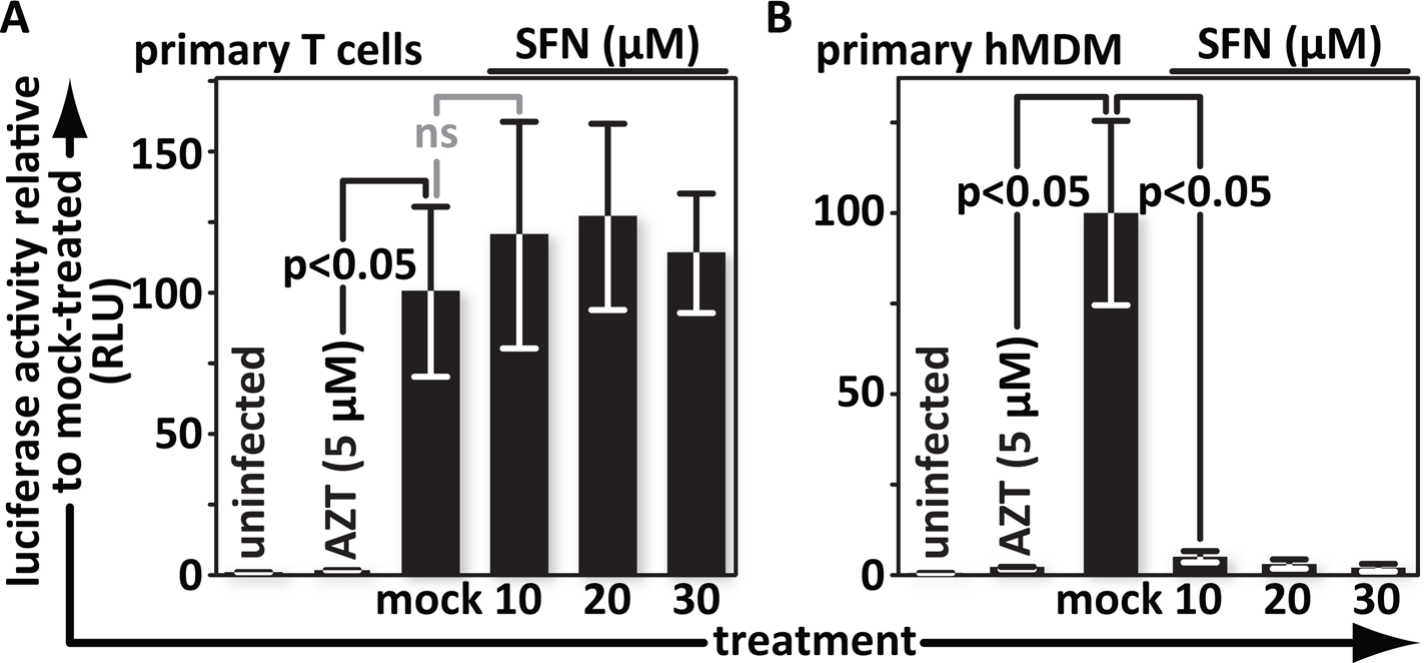
SFN blocks HIV in primary macrophages but not primary T cells. (A), Primary T cells and (B), hMDMs were treated with media supplemented with vehicle only (DMSO) or with 10 μM, 20 μM or 30 μM SFN. Pretreatment of cultures with 5 μM of the reverse transcription inhibitor zidovudine (AZT) served as a positive control for viral inhibition. Twenty-four hours after treatment, cells were either mock infected or infected with VSV-G-pseudotyped HIV-1, encoding firefly luciferase in place of *nef*. Twenty-four hours after infection, the cells were harvested and luciferase activity was measured by photon emission. The bar graphs represent the data for replicate experiments (n = 3).

SFN, in various contexts, has been found to impair bacteria, fungi and even viruses including Influenza A, respiratory syncytial virus (RSV) and Epstein-Barr virus (EBV) [35–39]. The impact of SFN on HIV infection however has not been tested in primary immune cells. Here, primary T cells were mock-treated or treated with 10 μM, 20 μM or 30 μM SFN for 24 hours. AZT-treated cells (5 μM) served as positive controls for infection inhibition. Cultures were infected with identical aliquots of VSV-G pseudotyped *env(‒)* HIV-1 luciferase reporter virus and harvested the following day. Cell lysates were tested for luciferase activity to quantify transcription from proviral DNA. While AZT suppressed infection, SFN did not impact infection throughout the dosage range tested (Fig 1A).

Dimethyl fumarate (DMF), a compound used to treat psoriasis and a known Nrf2 activator, slows HIV-1 spread in primary human monocyte derived macrophage (hMDM) cultures [40]. These experiments did not reveal how DMF interferes with HIV, but suggested to us that SFN could impact HIV infection in macrophages. This prompted us to test whether SFN blocks hMDM infection in single-round infection assays. Cells were mock treated or treated with 10 μM, 20 μM or 30 μM SFN for 24 hours as before. AZT-treated cells (5 μM) served as positive controls for infection inhibition. One day after infection with VSV-G pseudotyped reporter virus, culture lysates were assayed for luciferase activity. Here, unlike in primary T cell cultures, luciferase activity dropped sharply in cultures treated with 10 μM SFN and fell to background levels with higher SFN concentrations (Fig 1B). Thus, SFN acts to block single-round HIV-1 infections in primary hMDMs but not in primary T cells.

### SFN blocks HIV infection in monocytoid cell lines but does not increase SAMHD1 expression

**Fig 2:**
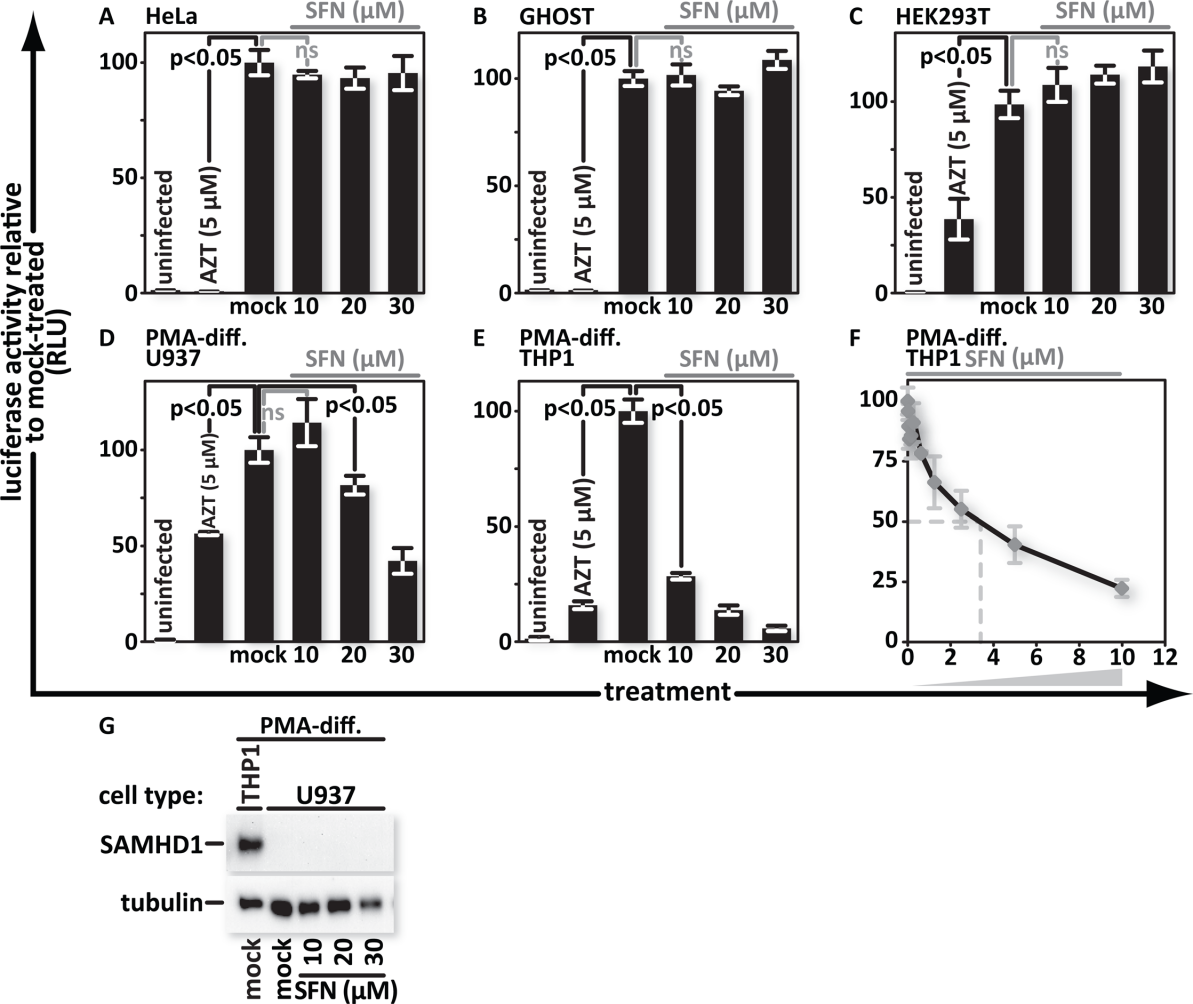
SFN blocks HIV infection in monocytoid cell lines but does not increase SAMHD1 expression. (A), HeLa, (B), GHOST, (C), HEK293T, (D), PMA-differentiated U937, and (E), PMA-differentiated THP1, cells were treated with media supplemented with vehicle only (DMSO) or with 10 μM, 20 μM or 30 μM SFN. Pretreatment of cell cultures with 5 μM AZT served as a positive control for viral inhibition. Twenty-four hours after treatment, the samples were infected with VSV-G pseudotyped HIV-1 encoding firefly luciferase in place of *nef*. Twenty-four hours after infection, the cultures were harvested and luciferase activity was measured by photon emission. (F), PMA-differentiated THP1 cells were treated with SFN that underwent a twofold serial dilution with 10μM of SFN being the highest concentration. Twenty-four hours after treatment, the samples were either mock infected or infected with VSV-G pseudotyped HIV-1 encoding firefly luciferase in place of *nef*. Twenty-four hours after infection, luciferase activity was measured. The bar graphs represent the data for replicate experiments (n = 3). (G), SAMHD1 protein was detected by western blot analysis from lysates of mock- and SFN treated PMA-differentiated U937 cells. Lysates from mock-treated, PMA-differentiated THP1 cells served as a positive control for SAMHD1 production.

To gain a better understanding of the breadth of SFN anti-viral action, we tested HeLa, GHOST and HEK293T cells, as well as monocytoid U937 and THP1 cells that were differentiated into a macrophage-like state by incubation in PMA-supplemented media. The cells were pretreated for 24 hours, then infected and assayed as described above. We detected no inhibition of HIV infection after SFN treatment in HeLa, HEK293T or GHOST cells (Fig 2A–2C) but SFN markedly decreased evidence of infection in U937 and THP1 cultures (Fig 2D and 2E). Further titration of SFN in cultures of PMA-differentiated THP1 cells shows that infections can be reduced by 50% when cultures are treated with SFN in the 2.5 to 5 μM range (Fig 2F).

U937 cells do not produce detectable levels of SAMHD1, an anti-viral protein that is expressed and active in PMA-differentiated THP1 cells and in hMDMs [41]. HeLa and HEK293T cells express SAMHD1, but its anti-viral function is inactivated by cell cycle kinase-mediated phosphorylation [42]. Here we asked whether SAMHD1 could be responsible for SFN-mediated anti-viral action in U937 cells. SFN blocks HIV replication in U937 cultures, albeit at higher concentrations than in those of primary macrophages or of THP1 cells (Fig 2D versus 2E). This raised the possibility that SFN triggers SAMHD1 production in U937 cells to block infection. We immunoblotted lysates of mock- and SFN-treated U937 cells for SAMHD1 and found that while SAMHD1 was readily detected in THP1 lysates, it remained undetectable in U937 lysates under the conditions tested (Fig 2G). This indicates that SFN does not block HIV by increasing SAMHD1 levels.

Importantly, SFN does not appreciably affect the viability of the cell-types tested. In order to determine whether cytotoxicity contributes to the apparent anti-viral action of SFN, we assessed cellular dehydrogenase activity in the presence or absence of SFN. Primary hMDMs and T cells, HeLa, GHOST and HEK293T cells as well as PMA-differentiated U937 and THP1 cells were treated with SFN for 24 hours as indicated. The translation inhibitor blasticidin served as a positive control for toxicity. Blasticidin-treated cells all showed decreased dehydrogenase activity; SFN however did not reduce dehydrogenase activity in most of the cell types tested. A slight reduction in dehydrogenase function was seen in THP1 cells at the higher concentrations tested (S1 Fig). Overall these data indicate that SFN mediated impairment of HIV infection is not due to decreased cell viability as indicated by sustained metabolic activity.

### The SFN-mediated HIV infection block is reversible

**Fig 3:**
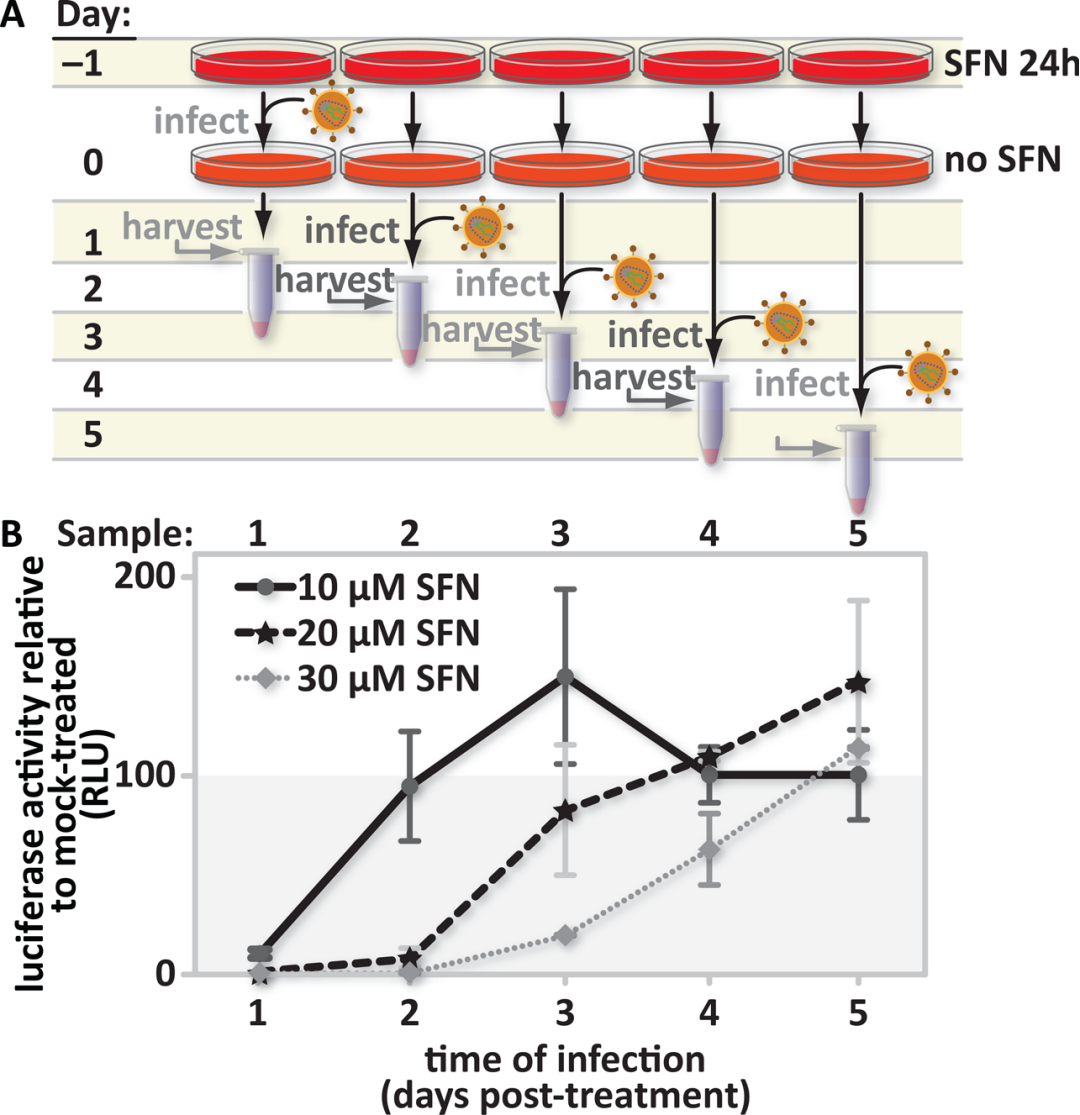
The SFN-mediated HIV infection block is reversible. (A), Study design: PMA-differentiated THP1 cells were pretreated with media supplemented with vehicle only (DMSO) or with 10 μM, 20 μM or 30 μM SFN. After twenty-four hours of treatment, the culture media was replaced with fresh SFN-free media. Cultures were infected 1, 2, 3, 4, or 5 days after treatment with freshly thawed aliquots of VSV-G pseudotyped HIV-1 encoding firefly luciferase in place of *nef*. Twenty-four hours after the start of each time-point infection, the cells were harvested and (B), luciferase activity was measured by photon emission. The bar graph represents the data for replicate experiments (n = 3).

SFN treatment could cause changes in cells that continue to impact HIV infection long after treatment. To address this possibility we tested the durability of the SFN-mediated anti-viral state in THP1-derived macrophages. All replicate cultures were treated with SFN for 24 hours. The SFN-containing media was then replaced with SFN-free media and new sets of cultures were exposed to freshly thawed aliquots of the same virus stock at 24-hour intervals. Each set of cultures was harvested for luciferase assays 24 hours after infection (Fig 3A). We found that infectability recovered over time in a dose-dependent manner (Fig 3B) with higher doses of SFN yielding a longer-lasting infection block. After removal from 10 μM SFN, cultures were as infectable as mock-treated controls in about 24 hours. These data show that the anti-viral effect induced by SFN treatment is reversible and that the SFN dose determines the duration over which the anti-viral state is maintained.

### SFN action impacts HIV-1 as well as HIV-2 and is not reporter dependent

**Fig 4:**
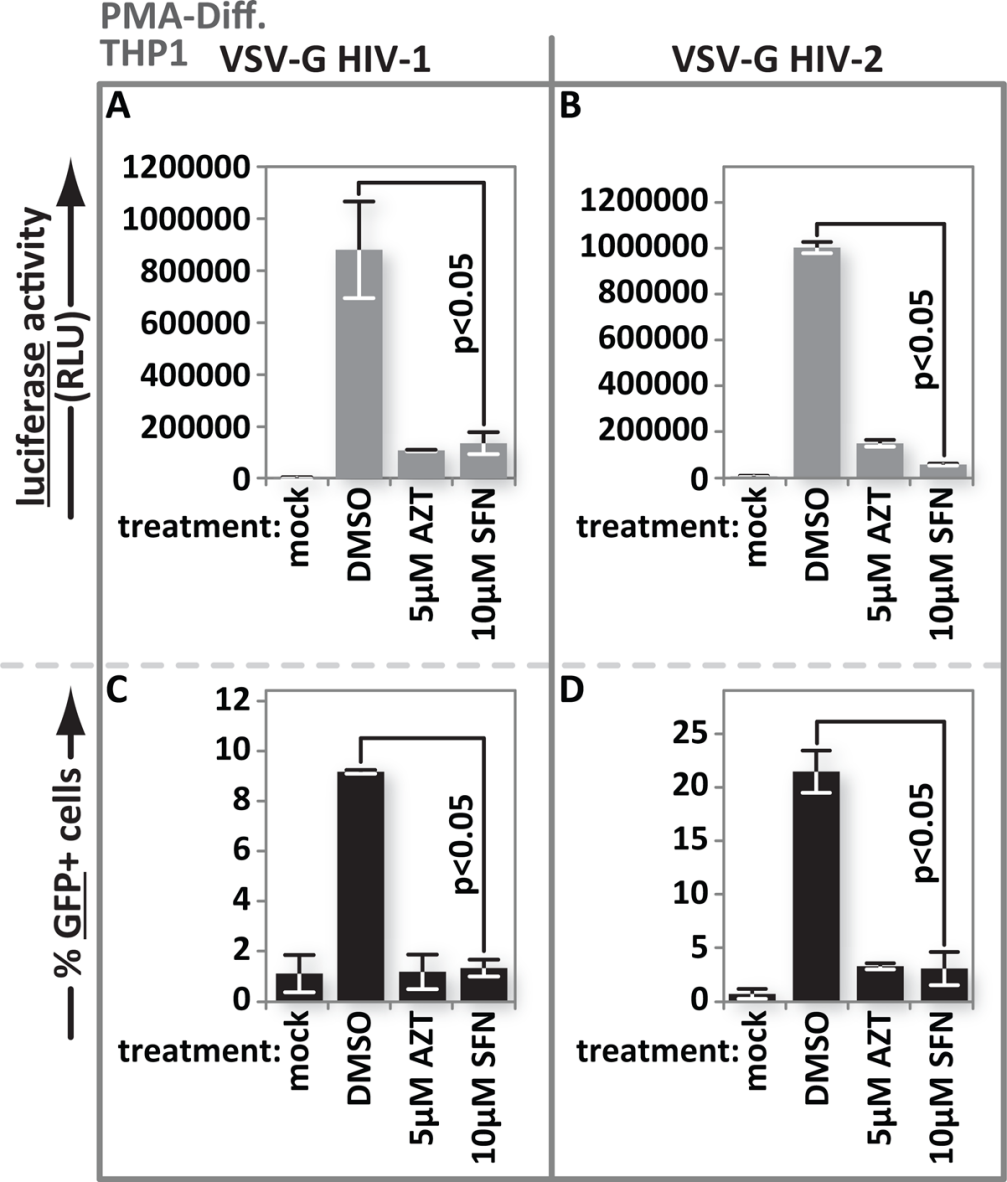
SFN action impacts HIV-2 as well as HIV-1 and is not reporter dependent. PMA-differentiated THP1 cells were treated with media supplemented with vehicle only (DMSO), 5 μM AZT or with 10 μM SFN. Twenty-four hours after treatment, the samples were either mock infected or infected with (A), VSV-G pseudotyped HIV-1 encoding firefly luciferase in place of *nef* or (B), VSV-G pseudotyped HIV-2 encoding firefly luciferase in place of *nef*. Twenty-four hours after infection, luciferase activity was measured by photon emission. (C), In parallel, the same experiment as in (A) and (B) was performed except that THP1 cells were infected with VSV-G-pseudotyped HIV-1 with GFP in place of *nef* or (D), VSV-G pseudotyped HIV-2 with GFP in place of *nef*. The samples with GFP-reporter viruses were fixed and harvested 24 h after infection and the fraction of GFP(+) cells was enumerated by flow cytometry. Bar graphs represent the data for replicate experiments (n = 3).

Here we tested whether SFN also impairs HIV-2 infection and whether SFN action is reporter-dependent. Cells were mock treated, or pretreated with vehicle, 5 μM AZT or 10 μM SFN, as indicated, and infected with VSV-G-pseudotyped HIV-1 or HIV-2 encoding luciferase in place of *nef* or with VSV-G-pseudotyped HIV-1 or HIV-2 encoding GFP in place of *nef*. Luciferase activity was measured in culture lysates and the percentage of GFP(+) cells was determined using flow cytometry. The loss of luciferase signal after SFN treatment in HIV-2-infected cultures paralleled that in the HIV-1-infected cultures (Fig 4A versus 4B). The decrease in the percentage of GFP(+) cells upon AZT or SFN treatment was also the same in HIV-1 and HIV-2 infected cultures (Fig 4A and 4B versus 4C and 4D). These results demonstrate that our observations, showing an SFN-mediated infection block, are not reporter-dependent. The similarity between the results for HIV-1 and HIV-2 further indicate that SAMHD1 is not responsible for the block because HIV-2 Vpx would be expected to at least partially overcome that restriction. The results also support a model, based on our flow cytometry data with the GFP reporter virus, in which fewer cells are becoming infected after SFN treatment, rather than one in which a similar number of infected cells produce less reporter protein.

### SFN action blocks spreading infections that rely on the HIV envelope for viral entry

**Fig 5:**
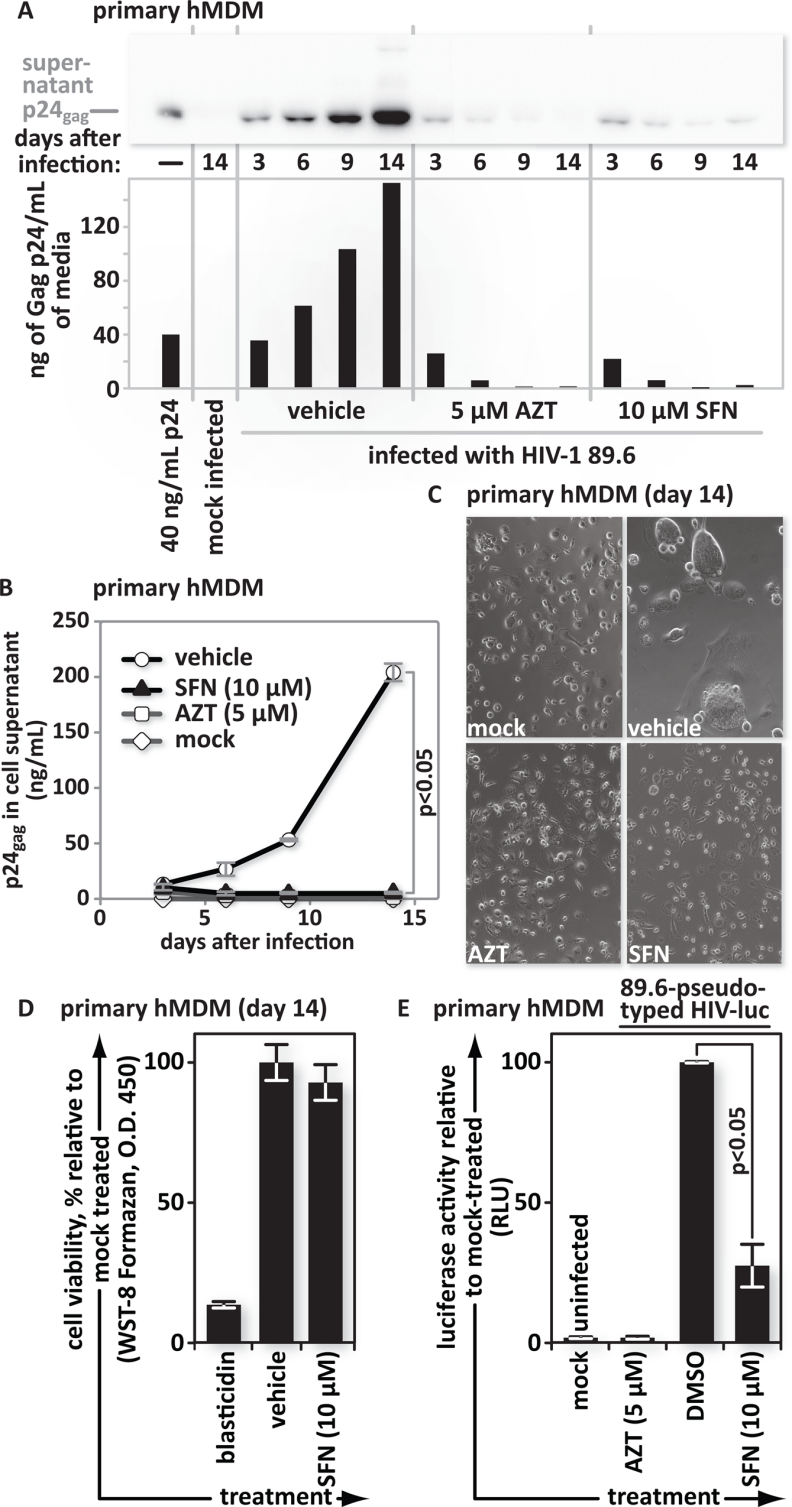
SFN action blocks spreading infections that rely on the HIV envelope for viral entry. hMDMs were pretreated with vehicle (DMSO), 5 μM AZT, or 10 μM SFN. All cultures were subsequently maintained in their respective treatments for the duration of the experiment. Twenty four hours after initial treatment, the cultures were infected with the HIV-1 clinical isolate 89.6. Culture supernatants were collected 3, 6, 9, and 14 days after infection. (A), Western blots and (B), p24 antigen ELISA assays of viral supernatants were performed. (C), Fourteen days after infection, cells were imaged using phase contrast microscopy. (D), Uninfected replicate cultures were maintained in the presence of vehicle (DMSO) or in 10 μM SFN. After 14 days of treatment, the viability of each cell type was assessed under each condition by measuring water-soluble tetrazolium salt (WST-8) formazan reagent cleavage by cellular dehydrogenases. Continuous treatment of cells with 10 μg/ml of the eukaryotic toxin blasticidin served as a positive control to demonstrate loss of viability. (E), hMDMs were infected with 89.6-Env-pseudotyped HIV-1 encoding firefly luciferase in place of *nef*. The bar graphs represent the data for replicate experiments (n = 3).

We exploited the characteristics of VSV-G-pseudotyped, *env(‒)* reporter viruses to determine that SFN pretreatment blocks infection at or before reporter production from integrated proviruses. Here we tested the impact of continuous SFN treatment on infections with the replication competent, reporter-free, dual-tropic HIV-1 strain HIV-1 89.6 [43]. Primary hMDMs were cultured in media containing DMSO, the vehicle for SFN, 5 μM AZT or 10 μM SFN. Culture supernatant samples were collected 3, 6, 9 and 14 days after infection and tested to determine the concentration of viral capsid protein, p24. Both western blotting and antigen-capture ELISA were employed. The concentration of viral capsid in the cell supernatant increased steadily in the vehicle control, however in the AZT- and 10 μM SFN-treated samples it decreased from residual input levels to background levels (Fig 5A and 5B). The HIV-1 89.6 Env glycoprotein can mediate fusion between adjacent macrophages, making inhibition of virus replication by SFN and AZT readily apparent in the cell cultures (Fig 5C).

Replicate cultures from these experiments were maintained in the presence of vehicle (DMSO) or 10 μM SFN for 14 days to determine the impact of SFN on cell health as measured by mitochondrial dehydrogenase activity. Blasticidin-treated cultures served as positive controls for viability loss. Continuous SFN treatment did not reduce cellular dehydrogenase activity relative to the vehicle only control (Fig 5D).

Finally, we tested whether SFN also stops virus bearing HIV-1 89.6 envelope glycoprotein on its surface in single-round infections. This was of course to determine whether virus with an HIV-1 envelope also encounters a restriction that blocks viral gene expression. As in the other single-round infections, we generated virus by transfecting HEK293T cells with a plasmid expressing the *env(‒)* luciferase reporter virus. Here, however we co-transfected with an HIV-1 89.6 envelope glycoprotein expression vector rather than one for VSV-G glycoprotein. As in the experiments with the VSV-G-pseudotyped virus, SFN significantly hindered infection with the HIV-1 89.6 envelope glycoprotein-pseudotyped virus (Fig 5E).

Overall, this set of experiments shows that SFN hinders both single-round and spreading infections. It further demonstrates that SFN interferes with infection of virus bearing the HIV envelope glycoprotein as it also interferes with VSV-G-pseudotyped virus. Finally, it also supports that our observation of SFN-mediated HIV inhibition is independent from exogenous reporter genes and shows that the SFN mediated block cannot be reversed by the Nef protein, which was absent from the luciferase and GFP reporter viruses.

### SFN acts through Nrf2 to block HIV infection of macrophages

SFN acts through Nrf2 to execute the majority of its known functions [44–46]. We thus hypothesized that the SFN-mediated block to HIV infection also relies on Nrf2. We first tested whether other known Nrf2 activators, dimethyl fumarate (DMF) and epigallocatechin gallate (EGCG), also block HIV in single-round macrophage infections. DMF has been shown to temper spreading infections in primary macrophages [40] but its impact on single-round infections has not been tested. EGCG inhibits Tat-induced LTR transactivation in HeLa cell-derived MAGI cells [47] but was not tested in macrophages. EGCG has also been reported to block engagement of the CD4 receptor by HIV-1 gp120 [40, 47, 48]. Here we use VSV-G pseudotyped HIV for single round infections to avoid possible contribution of this effect to our investigation of the impact of Nrf2 on HIV infection.

**Fig 6:**
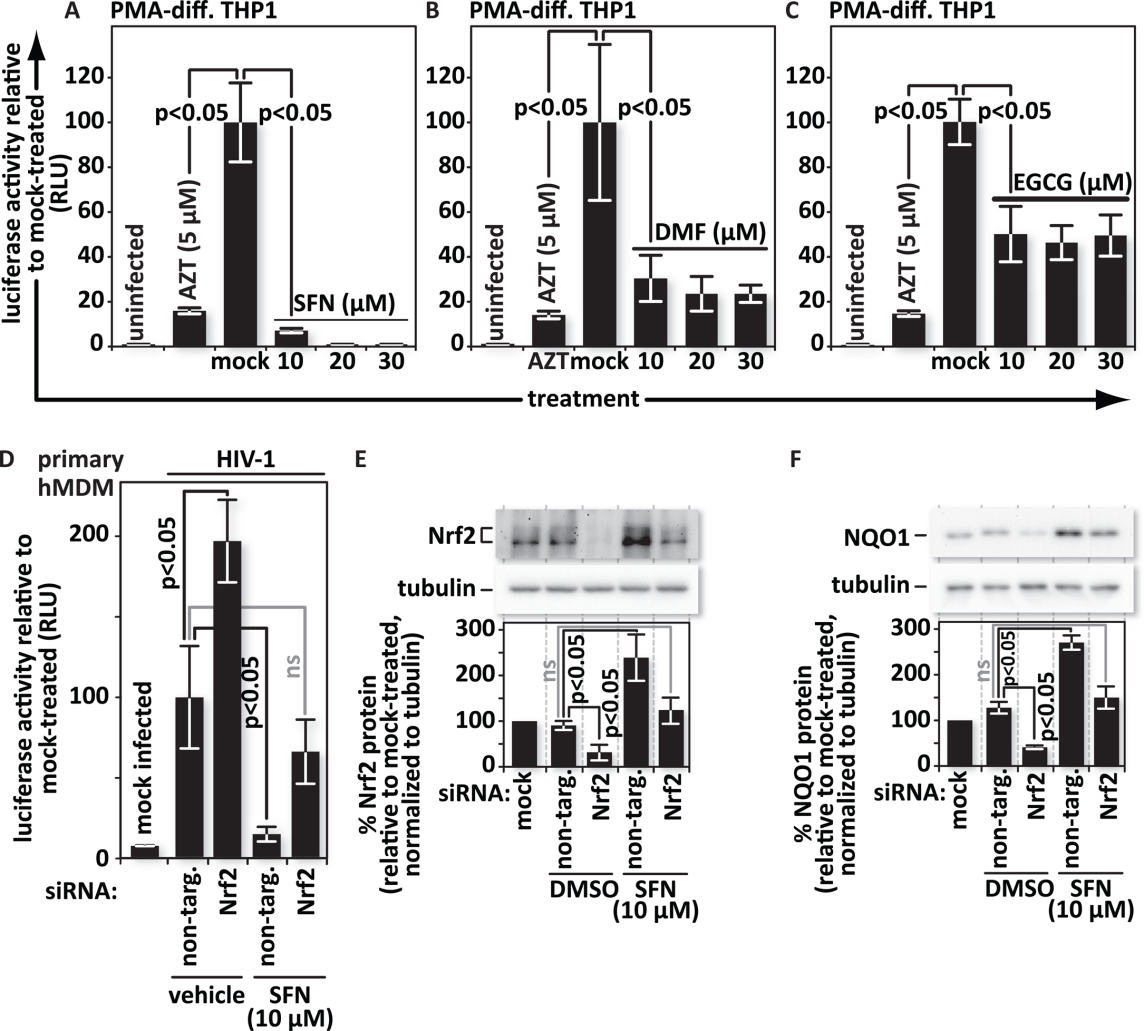
SFN acts through Nrf2 to block HIV infection in macrophages. PMA-differentiated THP1 cells were pretreated for twenty-four hours with the Nrf2 activators: (A), SFN, (B), DMF, or (C), EGCG at the indicated concentrations. Pretreatment of cultures with 5 μM of the reverse transcription inhibitor zidovudine (AZT) served as a positive control for viral inhibition. Twenty-four hours after treatment, cells were either mock infected or infected with VSV-G-pseudotyped HIV-1 encoding firefly luciferase in place of *nef*. Twenty-four hours after infection, the cells were harvested and luciferase activity was measured by photon emission. The bar graphs represent the data for replicate experiments (n = 3). (D), Cultures of hMDMs were transfected with either a non-targeting siRNA (control) or siRNA specific for Nrf2 mRNA. siRNA transfected hMDMs were either treated with vehicle (DMSO) or with 10 μM SFN. Twenty-four hours after treatment, the cells were either mock infected or infected with VSV-G-pseudotyped HIV-1 encoding firefly luciferase in place of *nef*. Twenty-four hours after infection, the cells were harvested and luciferase activity was measured by photon emission. The bar graphs represent the quantified data for replicate experiments (n = 3). (E) and (F), Representative samples from (D) were lysed and proteins from whole cell lysates were resolved by SDS-PAGE and identified by western blotting using antibodies with the indicated specificities. Densitometric analysis was performed on the Nrf2 and NQO1 (an indicator of Nrf2 function) bands and normalized to the values of the corresponding tubulin bands. The relative normalized intensities of the Nrf2 and NQO1 bands were then graphed. The data shown is representative of n = 3.

We found that like SFN, DMF and EGCG hinder reporter virus expression in single-round HIV-1 infections of PMA-differentiated THP1 cells and exhibit minimal toxicity (Fig 6A–6C and S2A–S2C Fig). This extends the previous work with DMF [40] by showing that the block is at a replication step at or before transcription from the provirus. Our observation that EGCG blocked VSV-G-pseudotyped virus eliminates the possibility that EGCG is thwarting a specific gp120/CD4 interaction in our system [48]. While we did not see SFN-mediated infection inhibition in HeLa cells (Fig 2A) we tested whether reporter expression from the provirus is inhibited by EGCG or the other compounds, as this could reflect inhibition of Tat function or transcription from the viral promoter. PMA-differentiated THP1 cells were pre-treated with AZT, DMSO, SFN, DMF or EGCG and then infected with VSV-G-pseudotyped luciferase reporter-encoding HIV-1 (S3A Fig). Replicate cultures were infected with the same reporter virus and, 5 days later, treated for twenty-four hours with DMSO, AZT, SFN, DMF or EGCG. While pretreatment with AZT, SFN, DMF or EGCG significantly inhibited luciferase activity, treatment after infection had much less or no impact. This suggests that each compound is likely acting before transcription in THP-1 infections, although we cannot rule out some impact after integration, especially for SFN. Neither DMF nor EGCG suppressed reporter activity to the same degree as SFN in pretreated cultures, perhaps because SFN is one of the most potent naturally occurring Nrf2 activators [49–52] or because SFN may also have an additional, albeit smaller, impact after proviral integration. These observations prompted us to test directly whether SFN blocks HIV-1 through Nrf2.

To determine whether SFN requires Nrf2 to impair HIV infection of macrophages, we depleted primary hMDMs of Nrf2 with siRNA and assessed the impact of this treatment on infection with VSV-G-pseudotyped HIV-1 luciferase reporter virus, both in the presence and absence of 10 μM SFN. In the absence of SFN, depletion of Nrf2 caused a significant increase in luciferase activity indicating that Nrf2 is detrimental to HIV infection in macrophages and that the basal level of Nrf2 that is present in macrophages is sufficient for establishing some anti-viral activity (Fig 6D). In cells transfected with non-targeting siRNA, SFN significantly reduced luciferase activity, reconfirming that SFN triggers a block to HIV infection of macrophages (Fig 6D).

SFN application, in combination with Nrf2-specific siRNA treatment, yielded a level of infectability that was lower than in the Nrf2-depleted, vehicle-treated cultures but higher than in cultures treated with non-targeting siRNA and SFN together. A western blot of parallel replicate cultures revealed that primary human macrophages express readily detectable quantities of Nrf2 (Fig 6E). Specific siRNA transfection reduced Nrf2 protein levels, but SFN treatment restored Nrf2 expression to levels seen in untreated cultures. Thus, SFN partially counteracted the impact of Nrf2-specific siRNA. Importantly however, Nrf2 levels remained inversely proportional to infection levels even in the absence of SFN. This supports a model in which HIV infection is countered specifically through Nrf2, and SFN acts to block HIV by increasing Nrf2 levels.

If Nrf2 triggers expression of a protein or an RNA with anti-viral function, then we also expect levels of the Nrf2-dependent protein NQO1 to be inversely proportional to infectability. As expected in this scenario, NQO1 levels fell below constitutive quantities upon siRNA-mediated depletion of Nrf2 and increased well above the base line in non-targeting siRNA-transfected cultures treated with 10 μM SFN. Consistent with our hypothesis, NQO1 levels in Nrf2-siRNA transfected cells returned to baseline upon treatment with 10 μM SFN (Fig 6F). Thus infection of hMDMs appears to be Nrf2-dependent. The loss of Nrf2 and Nrf2 function, as reflected by Nrf2-dependent gene expression, enhanced reporter activity. Conversely, the gain of Nrf2 function reduced reporter activity. Overall these data are consistent with an Nrf2-dependent block to HIV infection in hMDMs that is enhanced, here, by SFN treatment.

### SFN blocks infection after entry but before 2-LTR circle formation

**Fig 7:**
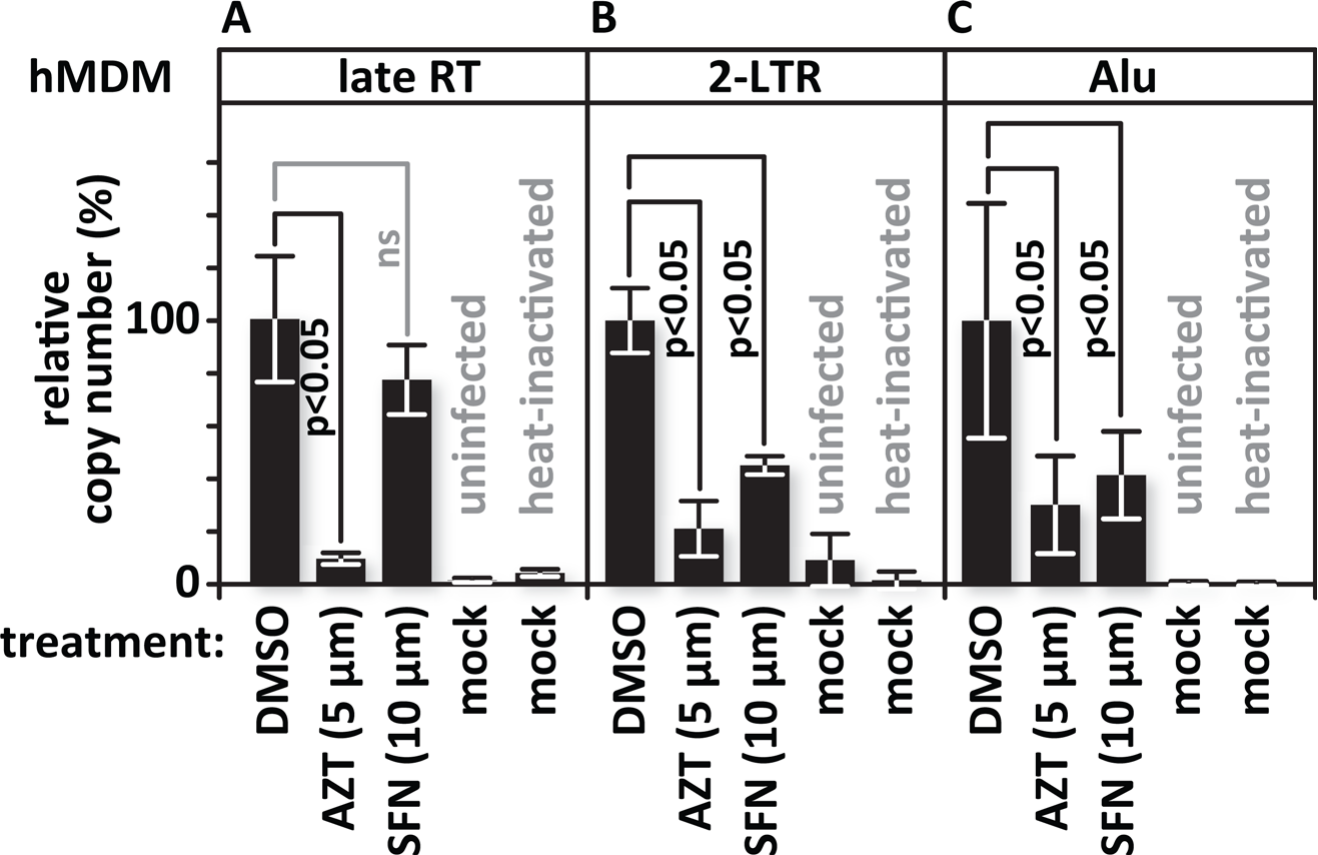
SFN blocks infection after entry and but before 2-LTR circle formation. Replicate cultures of hMDMs were pretreated with vehicle (DMSO)-containing media, with 5 μM AZT or with 10 μM SFN. Twenty four hours after treatment, the samples were infected with VSV-G-pseudotyped HIV-1 encoding GFP in place of *nef*. Cultures treated with heat-inactivated virus served as controls for plasmid carry over and for impaired viral entry. Cells were harvested and DNA was isolated 24 hours after infection. Viral DNA products were detected by real-time PCR using primer sets specific for the indicated stage of reverse transcription. (A), Relative quantities of late reverse transcription products, (B), 2-LTR circles, and (C), integrated proviruses. The bar graph represents the data for replicate experiments (n = 3).

SFN blocks single-round HIV-1 and HIV-2 infections (Fig 4). This limits the Nrf2-mediated block to steps that extend from attachment of the virus to the cell through transcription from the provirus. We found that SFN counters infection whether viral entry is mediated through HIV Env or VSV-G glycoproteins (Figs 1B versus 5E). This suggests a post-entry block as these glycoproteins engage different entry mechanisms. To further test whether SFN blocks after entry, we used real time PCR to quantify reverse transcription products in primary macrophage cultures infected in the presence or absence of SFN. We infected cells treated with vehicle, AZT or SFN with VSV-G-pseudotyped HIV-1 and quantified late reverse transcription products. Cultures exposed to the same quantity of heat-inactivated virus served to control for contamination of the viral stocks with the plasmid carrying the proviral clone used to generate the virus. The vehicle- and SFN-treated samples showed similar levels of late reverse transcription product, indicating that SFN, unlike AZT, did not hinder replication steps from viral entry through reverse transcription (Fig 7A). When we tested for 2-LTR circles, non-productive infection by-products that correlate with the transport of viral preintegration complexes into the cell nucleus, we found that these were clearly decreased in the AZT and SFN samples relative to the vehicle controls (Fig 7B). Alu-PCR, testing for integrated proviral sequences, also showed that these were similarly decreased in AZT- and SFN-treated cultures to levels that were significantly lower than in the vehicle-treated controls (Fig 7C).

### SFN does not trigger expression of the interferon-stimulated anti-viral factors SAMHD1 or MX2

**Fig 8:**
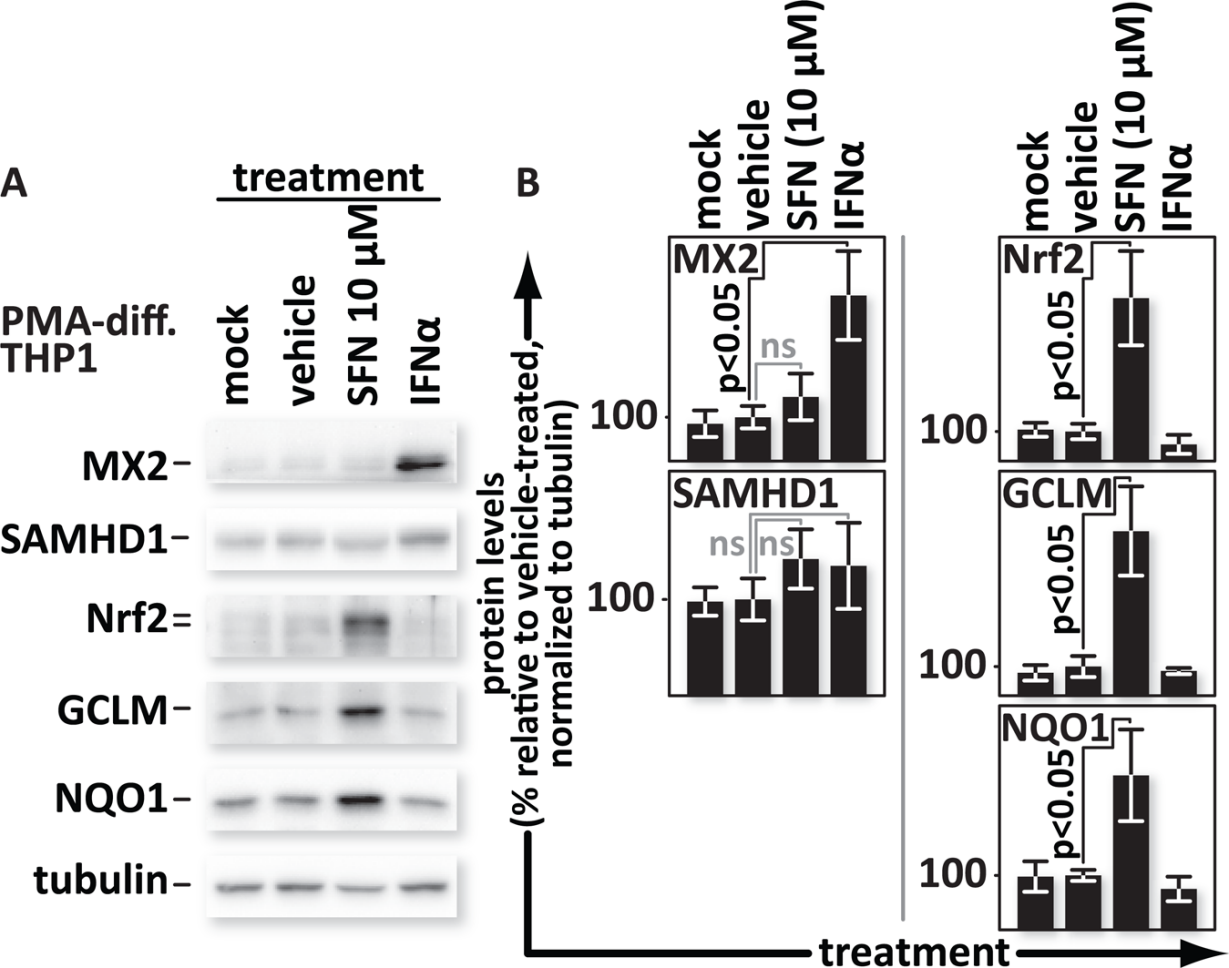
SFN does not trigger expression of interferon-stimulated anti-viral factors SAMHD1 or MX2. PMA-differentiated THP1 cells were mock-treated or treated with media supplemented with vehicle only (DMSO) or with 10 μM SFN or with 500 U/mL of IFNα. (A), Proteins from whole cell lysates were resolved by SDS-PAGE and identified by western blotting using antibodies with the indicated specificities. (B), Densitometric analysis was performed on the Nrf2, SAMHD1, MX2, NQO1 and GCLM (NQO1 and GCLM are both indicators of Nrf2 function) bands and normalized to the values of the corresponding tubulin bands. The relative normalized intensities of the bands were then graphed.

Dengue virus infection, in monocyte-derived dendritic cells, activates the IRF3/7/STAT1 and NF-κB anti-viral and inflammatory pathways, as well as Nrf2-dependent antioxidant genes. Interestingly, silencing of Nrf2 with siRNA increased innate anti-viral responses linked to interferon treatment [34]. Our experiments, with HIV in macrophages, sharply contrast this trend. Specifically, boosting Nrf2 counters infection while Nrf2 depletion supports infection. The restriction that we observed however, after reverse transcription but before 2-LTR circle formation, parallels those imposed by the type I interferon-inducible proteins SAMHD1 and MX2 [53, 54]. Here we asked whether SFN-mediated upregulation of Nrf2 protein levels causes an increase in the expression of those anti-viral proteins. We mock treated PMA-differentiated THP1 cultures or treated them with vehicle (DMSO) or 10 μM SFN, or with 500 U/mL of IFNα. As expected, SFN significantly increased levels of Nrf2 as well as of the Nrf2-regulated genes NQO1 and GCLM (Fig 8). SFN did not significantly change levels of either SAMHD1 or MX2. Conversely, IFNα treatment increased levels of MX2 as expected. SAMHD1 levels, despite some donor-to-donor variation, did not change significantly in response to IFNα, nor did the levels of Nrf2, NQO1 or GCLM. Thus, in our system, we do not see an overlap in the set of proteins that are upregulated by SFN and those that are upregulated by IFNα.

## Discussion

Here we have, for the first time, shown that SFN stops HIV infection in primary macrophages by triggering a block to infection that impacts the virus after reverse transcription but before 2-LTR circle formation. The SFN-regulated restriction relies on Nrf2, a transcription regulator of anti-oxidant genes. Importantly, we found that macrophages express sufficient Nrf2 to maintain a partial restriction even in the absence of SFN. This underscores that the anti-viral activity, while significantly enhanced by SFN through its augmentation of Nrf2 levels, does not require SFN to function.

Our work showing that *(1)* SFN blocks macrophage infection mediated by either VSV-G or HIV-1 Env, *(2)* SFN does not block infection in all cell-types and *(3)* blocks replication after late reverse transcription products have been formed, all support a model in which the restriction is well after entry into the cell but likely before the viral pre-integration complex is imported into the cell nucleus. The lack of anti-viral SFN action in numerous cell types and the dependence in macrophages solely on Nrf2 also indicates that SFN is neither inhibiting HIV directly by modifying a viral component nor is it modifying and thereby inhibiting a cellular component that the virus needs for successful infection. Similarly it is unlikely that SFN is activating a protein, post-translationally, that blocks HIV directly.

Our experiments comparing SFN action on GFP reporter virus with that on luciferase reporter virus further support a model in which the block is before the level of transcription. We used the GFP reporter virus to quantify individual infected cells and the fraction of those infected cells closely paralleled the population-wide reporter activity measured with the luciferase reporter virus. This indicates that the SFN-mediated block reduced the number of infected cells rather than suppressing transcription from similar numbers of infected cells.

HIV-1 Tat was reported to increase Nrf2 levels in HeLa-derived MAGI cells which corresponded with decreased Tat-mediated HIV-1 LTR transactivation [47]. While we did not test MAGI cells, SFN did not detectably impact LTR-directed luciferase reporter expression in HeLa or other non-macrophage-like cells. Our finding that SFN strongly inhibits infection at or before 2-LTR formation could account for the entire SFN-mediated block however, based on data shown in S3 Fig, we cannot fully rule out the possibility that SFN also impacts transcription from virus that evaded the initial block.

Our findings also put into perspective previous work that tested the neuroprotective effects of di- and monomethyl fumarate (MMF). This study showed that DMF and MMF can attenuate spreading HIV infections in hMDMs [40]. Our work, employing single-round infections, allowed us to determine more specifically that DMF hinders a process before expression of new viral proteins. DMF and MMF, like SFN, increase levels of Nrf2 [55], suggesting that these compounds also block HIV in macrophages through Nrf2. Interestingly, in our experiments SFN blocked infections more effectively at lower concentrations than DMF did [40].

None of the genes known to be modulated by Nrf2 direct production of recognized anti-viral products [28]. It is however possible that Nrf2 initiates a different expression pattern in macrophages than it does in previously tested B lymphocyte-derived cell lines [28]. In considering gene expression changes that could occur in human macrophages after SFN treatment, our attention was drawn to work describing murine macrophages treated with oxidized phospholipids [56]. These cells, designated Mox, adopt an Nrf2-dependent phenotype that is distinct from that of both M1 and M2 macrophages. Like SFN treated B lymphocyte-derived cells [28] or the THP1 cells in our work, and unlike M1 macrophages, Mox cells do not upregulate expression of known anti-viral genes. SFN treatment, on the other hand, may elicit a transcription pattern distinct from that described for Mox cells as we observed increases in both GCLM and NQO1 (Fig 8) while only GCLM was upregulated after treatment with oxidized phospholipids.

Future experiments, measuring the infectivity of heterokaryons formed by fusing cells that block HIV in response to SFN and those that don’t, will help to define the SFN-mediated block as a lack of a factor required for infection or the induction of a factor that blocks infection. This will help to guide the search for the mechanism underlying the SFN-mediated block. Presently however, the lack of HIV-associated factors among SFN-modulated genes is not surprising since new cellular anti-HIV factors and HIV co-factors continue to be identified [53, 54, 57–67]. We thus have discovered in SFN, a new way to activate an anti-viral state that HIV may not have previously encountered. It is therefore unlikely that the virus has developed an effective countermeasure.

SFN, and other Nrf2 stimulators, could be used therapeutically to block HIV infection of macrophages and possibly of other important HIV targets. The capacity to hinder both HIV-1 and HIV-2 provides an advantage over several protease inhibitors as well as the fusion inhibitor enfuvirtide and first generation non-nucleoside reverse transcription inhibitors which only block HIV-1 [68]. Further investigation will be required to determine why SFN fails to block infection in T cells, whether the same pathway could be exploited there as well or whether the restriction can be activated directly by other means.

Drugs based on their electrophilic potential, like SFN, have been widely used. These include β-lactam antibiotics [69] and drugs that generate electrophilic intermediates through metabolism like the heartburn medicine omeprazole, or clopidogrel which is used against strokes and heart attacks [70–74]. Afatanib, another such drug, was approved by the FDA for the treatment of lung cancer [75]. Beloranib, also an agent with an electrophilic center, is in trials for the treatment of obesity [76]. The isothiocyante group of SFN can be replaced with other groups to further optimize this compound as a therapeutic agent [77]. Work in this regard has shown that substituting the sulfoxide group of SFN with a ketone group resulted in a compound that retained the biological activity of SFN itself [78].

Finally, the discovery that Nrf2 mobilization counters HIV infection in macrophages stands in contrast with recent work showing that Marburg and Dengue virus as well as KSHV initiate and benefit from increased Nrf2 expression [30, 32, 34, 41]. All four viruses share tropism for cells within the myeloid lineage but KSHV, Dengue and Marburg virus, unlike HIV, also share the capacity to infect endothelial cells [79–81]. The consequences of how HIV, KSHV and Marburg virus interface with Nrf2 may be a reflection of this tropism difference, especially if the putative Nrf2-regulated anti-viral defense is not active in endothelial cells.

## Materials and Methods

### Ethics statement

All primary cells were obtained from de-identified donors at the University of Nebraska Medical Center under a category 4 exemption from consent procedures based on the anonymity of the donors. This exemption was approved by the Albany Medical College Committee on Research Involving Human Subjects.

### Cell culture

HEK293T (ATCC), HeLa (ATCC) and GHOST R5/X4 cells (from Dr. Dan Littman and Dr. Vineet Kewalramani, NIH AIDS Reagent Program, Division of AIDS, NIAID, NIH) were maintained in Dulbecco’s Modified Eagle Medium (DMEM) supplemented with 5% fetal bovine serum (FBS), 1 mM glutamine, 50 U/ml penicillin and 50 μg/ml streptomycin at 37°C and 5% CO_2_.

Monocytic THP-1 (ATCC) and U937 (ATCC) cell lines were cultured in Roswell Park Memorial Institute 1640 media (RPMI), with 5% FBS, 1 mM glutamine, 50 U/ml penicillin and 50 μg/ml streptomycin. These cells were differentiated into a macrophage-like state in media containing 5 ng/ml phorbol myristate acetate (PMA) for 5 days (37°C and 5% CO_2_).

Human lymphocytes were isolated from whole blood as buffy coats by Ficoll density gradient centrifugation. Monocytes were isolated by adhesion and differentiated into macrophages (hMDM) for 10–14 days in DMEM supplemented with 10% human AB serum. Primary T cells were maintained in DMEM supplemented with 10% human AB serum, 2.5 μg/ml phytohaemagglutinin (PHA), and 10 U/ml interleukin-2 at 37°C and 5% CO_2_. Half of the culture media was replaced with fresh media every 3 days.

### Virus preparation

HEK293T cells were transfected with virus expression vectors encoding either HIV-1: pNL4-3*env*(‒)*nef*(‒)*gfp*(+) (a gift from Dr. Vicente Planelles), pNL4-3*env*(‒)*nef*(‒)*luc*(+) (a gift from Dr. Nathaniel Landau), HIV-2 (pGL-AN*nef*(‒)*gfp*(+) a modified version of a clone provided by Dr. Mikako Fujita in which the gene for GFP was inserted in place of *nef*), HIV-2 *luc*(+) (a gift from Dr. Lee Ratner) or dual-tropic, full length HIV-1 89.6 (AIDS Reagent Program, Division of AIDS, NIAID, NIH: HIV-1 89.6 from Dr. Ronald Collman). Where specified, vectors carrying *env*(‒) proviral clones were co-transfected with pCL-VSV-G, a vesicular stomatitis virus G protein (VSV-G) expression vector. Standard calcium phosphate transfection was used. Viruses for spreading or single-round infections were harvested 48 hours after transfection of the proviral clones. Virus in each experiment was produced in the absence of anti-viral agents. Viral titers were determined by serial dilution on the GHOST R5/X4 indicator cell line.

### Reagents

Sulforaphane (SFN) (catalog no.: S8044, LKT Laboratories, Inc., St. Paul, MN) and Dimethyl Fumarate (DMF) (catalog no.: 242926, Sigma-Aldrich, St. Louis, MO) and epigallocatechin gallate (EGCG) (catalog no.: E4143, Sigma-Aldrich, St. Louis, MO) were dissolved in dimethyl sulfoxide (DMSO). Azidothymidine (AZT) (AIDS Reagent Program, Division of AIDS, NIAID, NIH Germantown, MD) and epigallocatechin gallate (EGCG) (catalog no.: G6817, LKT Laboratories, Inc., St. Paul, MN) were dissolved in water (50 mM stock).

### Luciferase assay

Luciferase activity was measured using the Promega Luciferase Assay System (Promega, Madison, WI) according to the manufacturer’s instructions. Culture medium was removed and cells were washed with phosphate-buffered saline (PBS). Samples were lysed and cellular debris was cleared by centrifugation at 18,000 × g for 1 minute. Twenty microliters of each sample were mixed with 80 μL of Luciferase Assay Reagent. Luciferase activity, in Relative Light Units (RLU), was measured using a Perkin Elmer VICTOR 3V 1420–040 multi-detection microplate reader.

### Cell viability

Cells were seeded at 10,000 per well in a 96-well plate (BD Labware, Franklin Lakes, NJ) in triplicate. The cells were then either mock-treated or treated with 10 μM, 20 μM or 30 μM SFN for 24 hours. Cell viability was assessed by measuring metabolic activity using a 2-(2-methoxy-4-nitrophenyl)-3-(4-nitrophenyl)-5-(2, 4-disulfophenyl)-2H-tetrazolium monosodium salt (WST-8) assay which produces a water-soluble formazan-dye upon reduction in the presence of an electron mediator (Cell Counting Kit 8, Dojindo, Kumamoto, Japan). The absorbance at 450 nm was measured with a microplate reader (BioTek, USA). Treatment of cells with 10 μg/ml of the eukaryotic toxin blasticidin served as a positive control for reduced viability.

### siRNA transfection

Short interfering RNAs (siRNA) were purchased from GE Life Sciences (Dharmacon): Non-targeting siRNA (catalog no.: P-002048-01-50) and Nrf2-specific siRNA (catalog no.: J-003755-09-0020). siRNA transfections were performed using Lipofectamine 2000 (Life Technologies, Grand Island, NY, catalog no.: 11668–019) according to the manufacturer’s instructions. siRNA transfection of hMDMs was performed three times with a recovery period of two days between transfections to ensure robust depletion of target mRNA.

### Microscopy

Spreading HIV-1 89.6 infections in hMDM cultures were monitored daily, using a Zeiss Axio Observer Z1 microscope, to determine the integrity of the cells. 14 days after infection, phase-contrast images were acquired using the ZEN2012 software by Zeiss.

### Flow cytometry

Flow cytometry was used to detect GFP production in cells infected with reporter viruses. Cells were fixed for 20 minutes at room temperature with 2% formaldehyde and washed with PBS. Ten thousand events were recorded with a FACScan flow cytometer (BD Biosciences, San Jose, CA). The data was analyzed using FlowJo v7.5 software (Tree Star Inc., Ashland, OR).

### Immunoblotting

Proteins from cells lysed with Laemmli buffer (50 mM Tris-HCl (pH 6.8), 2% SDS, 10% glycerol, and 0.1% bromophenol blue) were resolved by SDS-PAGE, transferred onto PVDF membrane (Millipore, Billerica, MA) and probed with the indicated antibodies. The primary antibodies used were: anti-tubulin (catalog no.: N-356, Amersham, GE Healthcare, Pittsburgh, PA), anti-Nrf2 (catalog no.: sc-13032, Santa Cruz Biotechnology, Inc., Dallas, TX) anti-SAMHD1 (catalog no.: GTX83687; GeneTex, Irvine, CA), anti-NQO1 (catalog no.: 3187S; Cell Signaling Technologies, Danvers, MA), anti-GCLM (catalog no.: GTX114075; GeneTex, Irvine, CA), anti-Mx2 (catalog no.: D0214; Santa Cruz Biotechnology, Inc., Dallas, TX) and anti-HIV-1 p24 (catalog no.: 183–H12-5C, obtained from Bruce Chesebro and Hardy Chen through the NIH AIDS Research and Reference Reagent Program). Chemiluminescent blot imaging was done with an Alpha Innotech FluorChem HD2 Imaging system. Densitometric analysis was performed using the ImageJ image analysis software from the NIH.

### Real-time PCR

Real-time PCR was performed, as described by Mbisa *et al*.[82], Yamamoto et al. [83], and Butler et al. [84] to determine where in the hMDM infection process HIV was blocked by SFN. Supernatant from virus producing cells was DNase treated (catalog no.: AM2238, TURBO DNase, Ambion, Life Technologies, Grand Island, NY) to minimize possible contamination from the proviral clones used to generate the virus. Cultures in which cells were incubated with heat inactivated (65°C for 10 minutes) virus served as controls for carryover of the proviral clones. Total DNA was isolated using a DNeasy kit (Qiagen, Germantown, MD) according to the manufacturer’s instructions. Sample DNA was amplified using TaqMan Universal PCR Master Mix (Thermo Fisher) reagent and the StepOnePlus Real-Time PCR System (Life Technologies, Grand Island, NY). The following primers were used:

**Late RT**. MH531: 5’- TGTGTGCCCGTCTGTTGTGT- 3’ [82].

MH532: 5’- GAGTCCTGCGTCGAGAGATC-3’ [82].

Probe LRT-P: 5’-(FAM)-CAGTGGCGCCCGAACAGGGA-(TAMRA)-3’ [82].

**1** ^**st**^ **round PCR**. First-Alu-F: 5’-AGCCTCCCGAGTAGCTGGGA-3’ [83]

First-Alu-R: 5’- TTACAGGCATGAGCCACCG-3’ [83]

First-gag-R: 5’- CAATATCATACGCCGAGAGTGCGCGCTTCAGCAAG-3’ [83]

**2** ^**nd**^ **round PCR.** Forward MH535: 5’- AACTAGGGAACCCACTGCTTAAG-3’ [83]

Reverse tag-R: 5’-CAATATCATACGCCGAGAGTGC-3’ [83]

Probe MH603: 5’-(FAM)-ACACTACTTGAAGCACTCAAGGCAAGCTTT-(TAMRA)-3’ [83]

MH535: 5’-AACTAGGGAACCCACTGCTTAAG-3’ [84]

MH536: 5’TCCACAGATCAAGGATATCTTGTC-3′ [84]

Probe MH603: 5’-(FAM)-ACACTACTTGAAGCACTCAAGGCAAGCTTT-(TAMRA)-3’ [84]

**Reference gene for RT and 2-LTR**. GAPDH forward 5’-GCATGGCCTTCCGTGTCCCC-3’

GAPDH reverse 5’- CCCTCCGACGCCTGCTTCAC-3’

Probe: 5’-(FAM)-GGTGGACCTGACCTGCCGTCTAGA-(TAMRA)-3’

### p24 antigen quantification

hMDMs were pretreated with AZT, SFN, or DMSO (vehicle control) for 24 hrs prior to infection with dual-tropic HIV-1 89.6. hMDM cultures were subsequently maintained in media supplemented with AZT, SFN, or DMSO for the duration of the experiment (14 days). Viral supernatants were harvested 3, 6, 9 and 14 days after infection and stored at ‒80°C. HIV-1 p24 antigen capture ELISA assays were performed on all samples according to manufacturer’s instructions (catalog no.: 5421, ABL Inc. Rockville, MD).

### Statistical analysis

The two-tailed Student’s t-test was used to determine statistical significance of differences between sample measurements.

## Supporting Information

S1 Fig SFN does not appreciably affect the viability of the cell-types tested. (A), Primary T cells, (B), hMDMs, (C), HeLa, (D), GHOST, (E), HEK293T, (F), PMA-differentiated U937 cells, (G), PMA-differentiated THP1 cells with media supplemented with vehicle only (DMSO) or with 10 μM, 20 μM or 30 μM SFN. (H), PMA-differentiated THP1 cells were treated with SFN that underwent a twofold serial dilution with 10μM of SFN being the highest concentration. Twenty-four hours after treatment, the viability of each cell-type was assessed under each condition by measuring water-soluble tetrazolium salt (WST-8) formazan reagent cleavage by cellular dehydrogenases. Pretreatment of cells with 10 μg/ml of the eukaryotic toxin blasticidin served as a positive control to demonstrate loss of viability. The bar graphs represent the data for replicate experiments (n = 3). All error bars reflect one standard deviation.
(TIF)

S2 Fig DMF and EGCG do not appreciably affect the viability of the cell-types tested. PMA-differentiated THP1 cells were treated with 0, 10μM, 20μM or 30μM (A) SFN, (B) DMF and (C) EGCG. Twenty-four hours after treatment, the viability of each cell type was assessed under each condition by measuring water-soluble tetrazolium salt (WST-8) formazan reagent cleavage by cellular dehydrogenases. Pretreatment of cells with 10μg/ml of the eukaryotic toxin blasticidin served as a control to demonstrate loss of viability. The bar graphs represent the quantified data for replicate experiments (n = 3). All error bars reflect one standard deviation.
(TIF)

S3 Fig EGCG does not inhibit Tat-mediated LTR activation in macrophages. PMA-differentiated THP1 cells were either (A) pretreated for twenty-four hours prior to or (B) treated five days after infection with SFN, DMF or EGCG. Cultures treated with the reverse transcription inhibitor zidovudine (AZT) served as positive controls for viral inhibition. Cells were either mock infected or infected with VSV-G-pseudotyped HIV-1 encoding firefly luciferase in place of *nef*. The pre-treated cells were lysed twenty-four hours after infection and the cells treated five days after infection were lysed twenty-four hours after treatment. Lysate luciferase activity was measured by photon emission. The bar graphs represent the data for replicate experiments (n = 3). All error bars reflect one standard deviation.
(TIF)
