# Supplementary figures and images for: Sulforaphane Inhibits HIV Infection of Macrophages through Nrf2
Source: PLoS Pathog. 2016 Apr 19;12(4):e1005581. doi: 10.1371/journal.ppat.1005581 (PMC4836681; doi:10.1371/journal.ppat.1005581)

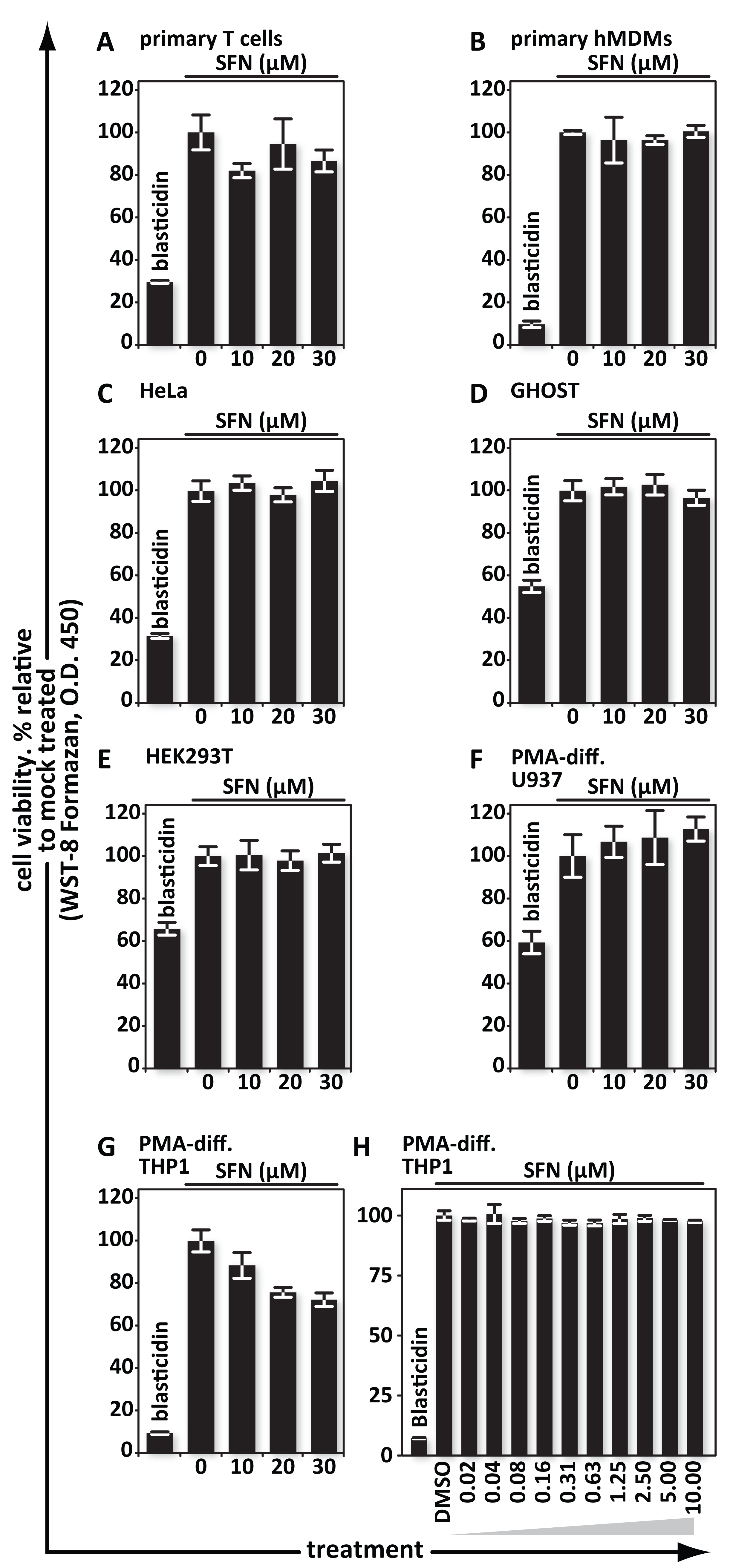

Supplement: S1 Fig — (A), Primary T cells, (B), hMDMs, (C), HeLa, (D), GHOST, (E), HEK293T, (F), PMA-differentiated U937 cells, (G), PMA-differentiated THP1 cells with media supplemented with vehicle only (DMSO) or with 10 μM, 20 μM or 30 μM SFN. (H), PMA-differentiated THP1 cells were treated with SFN that underwent a twofold serial dilution with 10μM of SFN being the highest concentration. Twenty-four hours after treatment, the viability of each cell-type was assessed under each condition by measuring water-soluble tetrazolium salt (WST-8) formazan reagent cleavage by cellular dehydrogenases. Pretreatment of cells with 10 μg/ml of the eukaryotic toxin blasticidin served as a positive control to demonstrate loss of viability. The bar graphs represent the data for replicate experiments (n = 3). All error bars reflect one standard deviation. (TIF) [file ppat.1005581.s001.tif]

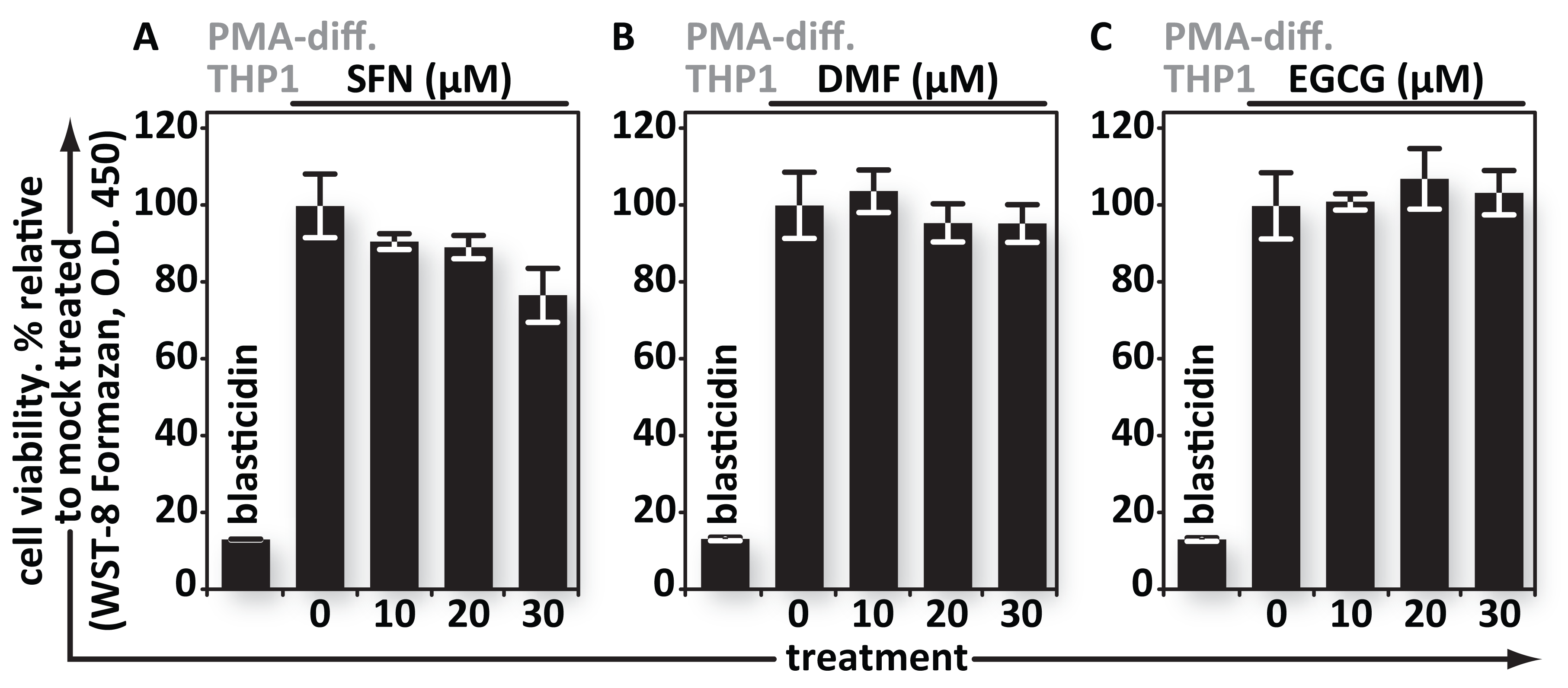

Supplement: S2 Fig — PMA-differentiated THP1 cells were treated with 0, 10μM, 20μM or 30μM (A) SFN, (B) DMF and (C) EGCG. Twenty-four hours after treatment, the viability of each cell type was assessed under each condition by measuring water-soluble tetrazolium salt (WST-8) formazan reagent cleavage by cellular dehydrogenases. Pretreatment of cells with 10μg/ml of the eukaryotic toxin blasticidin served as a control to demonstrate loss of viability. The bar graphs represent the quantified data for replicate experiments (n = 3). All error bars reflect one standard deviation. (TIF) [file ppat.1005581.s002.tif]

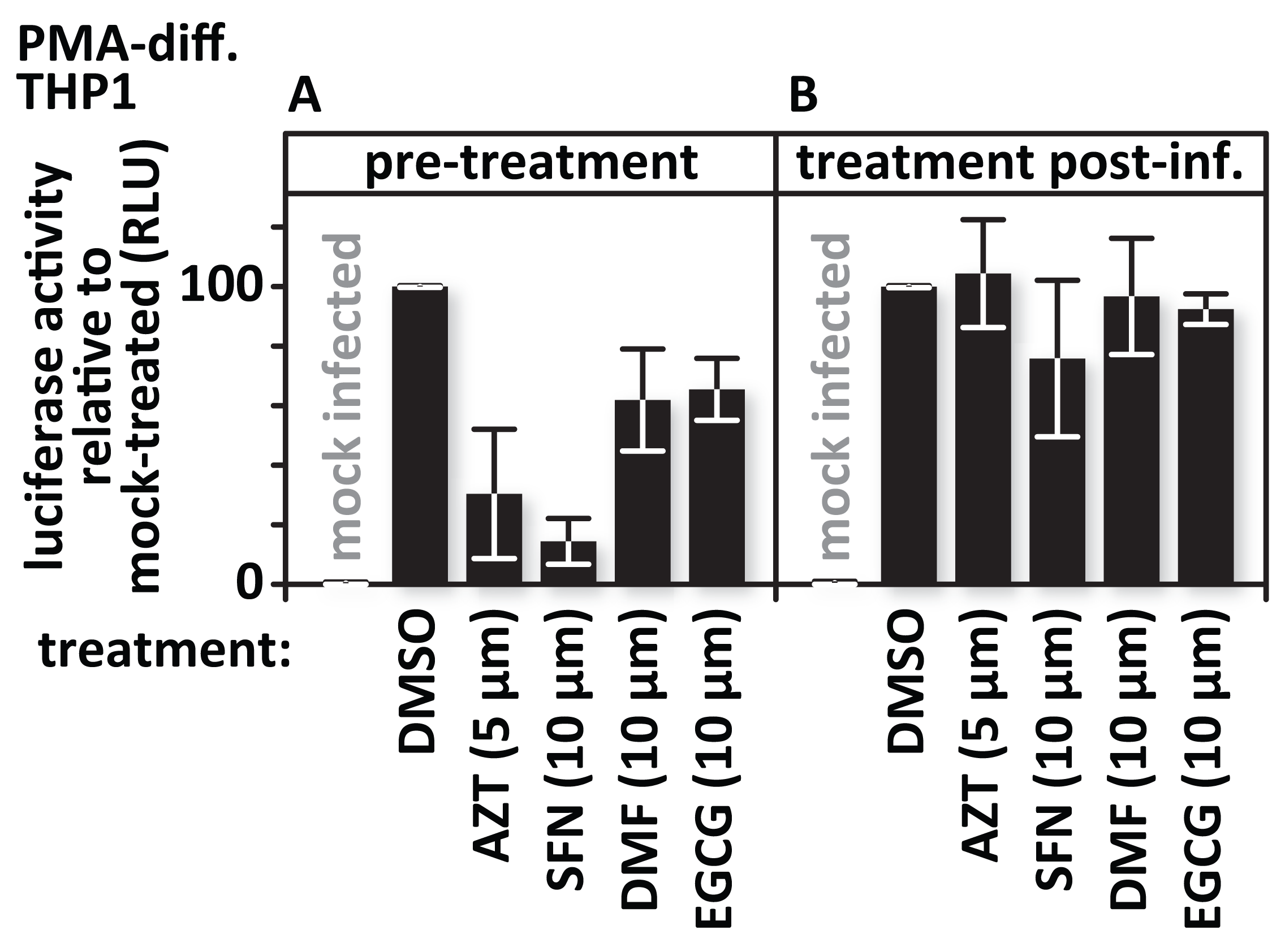

Supplement: S3 Fig — PMA-differentiated THP1 cells were either (A) pretreated for twenty-four hours prior to or (B) treated five days after infection with SFN, DMF or EGCG. Cultures treated with the reverse transcription inhibitor zidovudine (AZT) served as positive controls for viral inhibition. Cells were either mock infected or infected with VSV-G-pseudotyped HIV-1 encoding firefly luciferase in place of nef. The pre-treated cells were lysed twenty-four hours after infection and the cells treated five days after infection were lysed twenty-four hours after treatment. Lysate luciferase activity was measured by photon emission. The bar graphs represent the data for replicate experiments (n = 3). All error bars reflect one standard deviation. (TIF) [file ppat.1005581.s003.tif]
